# Finite Volume Scheme for Double Convection-Diffusion Exchange of Solutes in Bicarbonate High-Flux Hollow-Fiber Dialyzer Therapy

**DOI:** 10.1155/2012/973424

**Published:** 2012-10-31

**Authors:** Kodwo Annan

**Affiliations:** Department of Mathematics and Computer Science, Minot State University, Minot, ND 58707, USA

## Abstract

The efficiency of a high-flux dialyzer in terms of buffering and toxic solute removal largely depends on the ability to use convection-diffusion mechanism inside the membrane. A two-dimensional transient convection-diffusion model coupled with acid-base correction term was developed. A finite volume technique was used to discretize the model and to numerically simulate it using MATLAB software tool. We observed that small solute concentration gradients peaked and were large enough to activate solute diffusion process in the membrane. While CO_2_ concentration gradients diminished from their maxima and shifted toward the end of the membrane, HCO_3_^−^ concentration gradients peaked at the same position. Also, CO_2_ concentration decreased rapidly within the first 47 minutes while optimal HCO_3_^−^ concentration was achieved within 30 minutes of the therapy. Abnormally high diffusion fluxes were observed near the blood-membrane interface that increased diffusion driving force and enhanced the overall diffusive process. While convective flux dominated total flux during the dialysis session, there was a continuous interference between convection and diffusion fluxes that call for the need to seek minimal interference between these two mechanisms. This is critical for the effective design and operation of high-flux dialyzers.

## 1. Introduction

High-flux dialyzer is one of the possible treatments to remove toxic solutes from the blood when the native kidneys loss their function. Small solutes removal is primarily done by diffusion while larger solutes removal is obtained by convection. The efficiency of a dialyzer is therefore dependent on its ability to use these mechanisms (convection and diffusion) to exchange solutes across the dialyzer membrane [1–4]. Diffusion is mainly affected by blood and dialysate flow rates, dialyzer surface area, temperature, and membrane thickness. If we assume constant values to all other factors, then the diffusion mechanism depends on the blood and dialysate concentration gradients [5, 6]. This, however, is influenced by the blood and dialysate flow distributions and flow rates. Extensive research has been done on flow distribution mismatch frequently observed at the blood-dialysate interface [6–10]. Physically, attempts to correct and optimize blood and dialysate flow mismatch have been made by redesigning blood and dialysate headers. Options such as space yarns and moiré structure have been proposed to resolve dialysate channeling phenomenon external to the fiber bundle [11, 12]. The main feature of convection is the use of high-flux HD characterized by high permeability for water, electrolytes, and higher clearance of middle and large molecular weight solutes. The role of convective transport is discussed extensively in recent articles [13–19]. 

The investigation of the effect of convection and diffusion during dialysis session continues to pose a major challenge to HDF researchers and engineers. Best-known earlier models were based on clinical data. However, these macroscopic experimental approaches make it difficult to capture and explore convective and diffusive transports during dialysis session. Mathematical models have been used to evaluate, optimize, and control various forms of dialysis therapy from clinical routine to investigating new issues in dialysis therapy [3–6, 8–11, 15, 19, 20]. The underlying mechanism of these mathematical models has been Navier-Stoke equation. Numerical methods aimed to quantify both the convective and diffusive transports of solutes exchange across membranes have been used [21–28]. Researchers [21–23, 25, 27, 28] used finite difference schemes and control volumes while analytical solutions were derived by [24, 26]. These authors, however, neglected the effects of either diffusion or convection flows, or their choice of techniques not justified in dialysis therapies, or did not include buffer which is common in dialysis sessions.

In dialysis, the type of numerical scheme used in computing solutions of convective-diffusive equations is very necessary, especially when high flux membrane (i.e., where convection term dominates) is used. In this case, the dialyzer membrane is so thin that one is forced to use under resolved methods that may be unstable. On the other hand, the use of dispersive schemes may trigger numerical instabilities which may affect the fully description of the convection and diffusion phenomena during HD session. To achieve maximal dialyzer efficiency, the accuracy and reliability of numerical schemes used to compute convection-diffusion phenomena is of paramount importance. These numerical schemes depend on the choice of discretization and the quality of the underlying mesh.

This paper focused on finite volume method (FVM) for unsteady-state convection-diffusion equations that arise in dialysis therapy. The transport equation was described using a three-compartmental model of blood, membrane, and dialysate compartments. The model was coupled with buffering and replenishment. An accurate transient convection-diffusion model that described solute exchange in a typical high-flux hollow-fiber dialyzer was performed. The numerical discretization and analysis schemes were then proposed and tested. We then explored the impact of small molecule weight solute (carbon dioxide and bicarbonate) transports in a high-flux dialyzer followed by conclusions.

## 2. Model Formulation

### 2.1. Membrane Model

The conservation law for the transport of solute concentrations in an unsteady flow has the general form

(1) $$\begin{array}{c} \overset{\text{Unsteady}\;\text{Term}}{\overset{︷}{\frac{\partial \rho c}{\partial t}}}+\overset{\text{Convection}\;\text{Term}}{\overset{︷}{\mathrm{div}\left(\rho cu\right)}}=\;\;\overset{\text{Diffusion}\;\text{Term}}{\overset{︷}{\mathrm{div}\left(D\cdot \text{grad}\left(c\right)\right)}}\;\;+\overset{\text{Source}\;\text{Term}}{\overset{︷}{S_{c}}}, \end{array}$$

where *ρ* (a constant) is the density of incompressible fluid, *c* is the solute concentration, **u** is the fluid velocity, *S*_*c*_ is the production of new solute at that point, *D* is the diffusion constant, and div⁡() and grad() are the normal vector operators. Equation (1) basically states that the rate of increase of the number of molecules of solute (*ρc*) at any point equals the (negative of the) rate they are being removed at that point by convection (div⁡{*ρc* **u**}) plus the rate they are being added by diffusion (div⁡{*D*  grad(*c*)}) plus the rate at which solutes are being produced (*S*_*c*_). 

The following simplifying assumptions are made in the membrane model.

(1) The membrane is small enough that it is assumed to be in equilibrium (steady state), so the time derivative term is zero.
(2) The membrane impedes flow in all directions but radially, so the velocity vector is in the *r* direction only.
(3) The membrane volume is small that production of new solute can be ignored, so *S*_*c*_ = 0.
(4) Since we are interested in the change of concentration from one side of the membrane to the other, the rate of change of concentration in the axial direction is smaller (and it is zero in the *ϕ* direction due to symmetry). Therefore, we ignore the terms in grad(*c*) except for the radial direction. Using assumptions 1–4, (1) becomes
  (2) $$\begin{array}{c} \rho \cdot \mathrm{div}\left(cu\right)=D\cdot \mathrm{div}\left(\text{grad}\left(c\right)\right). \end{array}$$

Integrating (2) and transforming the resulting equation using the divergence theorem gives

(3) ρ∫CVdiv⁡(cu)dV=D∫CVdiv⁡(grad(c))dV,ρ∮F(n·cu)dA=D∮F(n·grad(c))dA,

where the normal vector to the face is **n**, and the integrals indicate either a volume integral over the control volume (with *CV*) or a surface integral over the faces (with *F*). All components of **u** in (3) are zero except the radial direction, so the dot product with **n** has only the term *cu*_*r*_. Using assumption 4 and assuming that the functions in the integrands are constant across the faces that the surface integrals act on, (3) reduces to

(4) ρur[c(r+δr2)−c(r−δr2)]  =D[∂c∂r(r+δr2)−∂c∂r(r−δr2)].

From (4), if the fluid velocity was zero, the LHS would be zero, the gradient would be a constant, and the concentration would follow a linear slope through the membrane in the radial direction. However, with a nonzero velocity, the flux of solute due to convection, *cu*_*r*_, is nonzero. Since the concentration varies through the membrane in the radial direction, intuitively, the diffusion term would need to compensate for the drop in convection so that a constant flow exists across the membrane from one side to the other. Re-arrange the terms in (4)

(5) ρurc(r+δr2)−D∂c∂r(r+δr2)  =ρurc(r−δr2)−D∂c∂r(r−δr2).

Since (5) is true for any separation *dr* and for any value *r*, the total solute flux, *J*_*s*_ (both convection and diffusion) is constant across the membrane at any point. Thus, we have

(6) $$\begin{array}{c} \rho u_{r}c\left(r\right)-D\frac{\partial c}{\partial r}\left(r\right)=J_{s}. \end{array}$$

The exact solution for (6) in terms of the concentration *c*(*r*) is of the form

(7) $$\begin{array}{c} c\left(r\right)=k_{1}e^{k_{2}r}+k_{3}. \end{array}$$

Substituting (7) into (6) and gathering terms

(8) ρurc(r)−D∂c∂r(r)=ρurk1ek2r+ρurk3−Dk1k2ek2r=k1(ρur−Dk2)ek2r+ρurk3=Js.

The coefficient of the exponential must be zero and the constant term equal *J*_*s*_, thus

(9) $$\begin{array}{c} k_{2}=\frac{\rho u_{r}}{D},\;\;k_{3}=\frac{J_{s}}{\rho u_{r}}. \end{array}$$

The constant *k*_1_ is determined by the concentration value at one end of the membrane. For this paper, the pressure in the dialysate side (at larger values of *r*) is normally larger than that in the blood side, so the velocity *u*_*r*_ is negative. Picking a solute where the concentration is higher in the dialysate side, gives positive concentration gradient, *dc*/*dr*. Therefore, if both terms of (6) are negative, then *J*_*s*_ is negative, that is, a total flux in the negative direction toward the blood side. In this case, *k*_3_ is positive, *k*_2_ is negative, and for *dc*/*dr* to be positive *k*_1_ must be negative. So the concentration function must be of the form

(10) $$\begin{array}{c} c\left(r\right)=\alpha_{3}-\alpha_{1}e^{-\alpha_{2}r}, \end{array}$$

where the *α*'s are the positive versions of the *k* constants. Therefore, concentration is positive and decreasing for smaller *r* (toward the blood side) and the slope is increasing for smaller *r* to compensate for the reduced convection.

### 2.2. Transmembrane Flow

**Figure 1:**
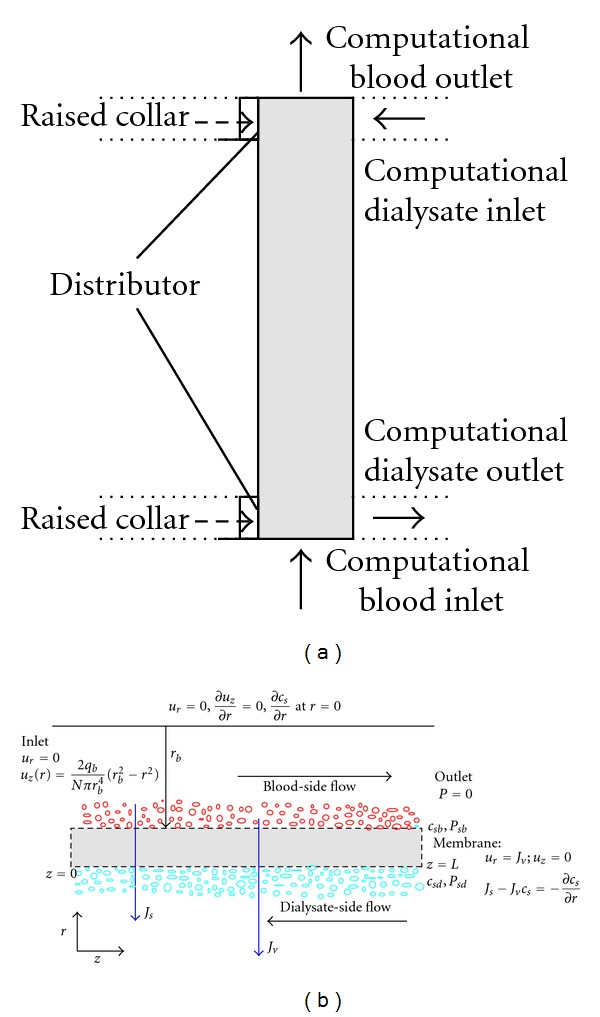
(a) Schematic of a typical hollow-fiber dialyzer module with the computational blood and dialysate domains. (b) Mass transport of solutes in blood and dialysate compartments through a single hollow fiber membrane.

Following [29] and assuming that reflection coefficient is negligible because of small (10^−4^) fiber pore size [30], we describe the flow passing through the membrane (see Figure 1(b)) by simplified Kedem-Katchalsky (K-K) equations

(11) Jv≈LpΔP,Js≈Cs∗Jv+PsΔcs.

*J*_*v*_ (m/s) is ultrafiltration velocity or volumetric flux across the membrane; *J*_*s*_  (kg/m^2^s) is solute flux across the membrane; *L*_*p*_  (m/sPa) is the hydraulic permeability of the membrane; *P*_*s*_  (m/s) is solute diffusive permeability coefficient of a membrane; *c*_s_*  (kg/m^3^) represents the average solute concentration at each side of the membrane; Δ*c*_*s*_  (kg/m^3^) is solute concentration difference (i.e., transmembrane concentration) across the membrane. The parameter Δ*P*  (Pa) is the membrane surface hydraulic permeability of the membrane. Thus, the membrane interfacial conditions for the blood-side model are

(12) $$\begin{array}{c} u_{z}=0,\;\;u_{r}=J_{v},\;\;D_{s}\frac{\partial c_{s}}{\partial r}=J_{v}c_{s}-J_{s}. \end{array}$$

### 2.3. Blood-Side Flow Model

Consider (*r*, *z*) as coordinates representing a point in the cylindrical coordinate system where the *z*-axis is taken along the dialyzer length (i.e., 0 ≤ *z* ≤ *L*) and *r* is taken along the radial direction. An axisymmetric domain, where *r* is chosen to lie in the range 0 < *r* < *r*_*b*_ between *z* = 0 and *z* = *L* for a membrane length *L* and radius *r*_*b*_  (see Figure 1) depicts the blood side model. The Navier-Stokes and continuity equations that govern the flow of an incompressible Newtonian fluid representing blood with constant density *ρ* and viscosity *μ* can be described as [13]

(13) 1r∂(rur)∂r+∂uz∂z=0,ur∂ur∂r+uz∂ur∂z=−1ρ∂p∂r+μρ[1r∂∂r(r∂ur∂r)−urr2+∂2ur∂z2],ur∂uz∂r+uz∂uz∂z=−1ρ∂p∂z+μρ[1r∂∂r(r∂uz∂r)+∂2uz∂z2],

where *u*_*r*_ and *u*_*z*_ are the radial and axial velocity components, respectively, and *p* the pressure. Using the continuity equation and the fact that flow is driven by pressure gradient in the *z*-direction, a fully developed inlet velocity profile for *N* number of fibers at *z* = 0 and 0 < *r* < *r*_*b*_ are obtained [31, 34]

(14) $$\begin{array}{c} u_{r}\left(r\right)=0,\;u_{z}\left(r\right)=\frac{2q_{b}}{N\pi r_{b}^{4}}\left(r_{b}^{2}-r^{2}\right). \end{array}$$

Here, *q*_*b*_ is the inlet blood flow rate in each of the hollow fibers with a fiber cross-section area *πr*_*b*_^2^. Applying no slip condition at the wall and axisymmetric axis, respectively, at *r* = 0

(15) $$\begin{array}{c} u_{r}=u_{z}=0,\;\;u_{r}=\frac{\partial u_{z}}{\partial r}=0\;\text{at}\;r=0;\;\;0\leq z\leq L. \end{array}$$

The convection-diffusion equation governing the mass transport of solutes *s* coupled to the blood velocity field is given by

(16) ∂cs∂t︷Transient  Term+  uz∂cs∂z+ur∂cs∂r︷Convective  Term  =  Ds(∂2cs∂r2+1r∂cs∂r+∂2cs∂z2)︷Diffusive    Term+Bs︷Buffer    Term,

where *c*_*s*_ and *D*_*s*_ are the concentration and the diffusion coefficient of solute *s* in the blood, respectively. The inlet and outlet boundary conditions for the concentration equation (4) are

(17) $$\begin{array}{c} c_{s}\left(z,r,0\right)=c_{s_{0}},\;\;c_{s}\left(0,r,t\right)=c_{s_{0}},\;\;\frac{\partial c_{s}\left(z,0,t\right)}{\partial r}=0. \end{array}$$

*B*_*s*_ defines the buffer term that vanishes everywhere except in the blood membrane domain and denotes the rate of solute *s* production or consumption per time. We adapt buffer reaction rates for *s* = [CO_2_, HCO_3_^−^] given by [31, 35]

(18) $$\begin{array}{c} B_{\text{C}{\text{O}}_{2}}=-k_{+}\left(1+\alpha \frac{2\left[{\text{C}{\text{O}}_{3}}^{2-}\right]}{\left[{\text{HC}{\text{O}}_{3}}^{-}\right]}\right)\left(\left[\text{C}{\text{O}}_{2}\right]-\beta \frac{\left[{\text{HC}{\text{O}}_{3}}^{-}\right]}{\left[{\text{C}{\text{O}}_{3}}^{2-}\right]}\right),\\ B_{{\text{HC}{\text{O}}_{3}}^{-}}=-2B_{\text{C}{\text{O}}_{2}}, \end{array}$$

where *α* = *k*_2_ *K*_4_/2*k*_+_ *K*_3_,  *β* = *k*_−_ *K*_3_/*k*_+_, and  [HCO_3_^−^]/[CO_3_^−2^] = 20. The rate controlling reactions for the carbonate and bicarbonate ions are given as [31, 35]

(19) $$\begin{array}{c} \text{C}{\text{O}}_{2}+{\text{H}}_{2}\text{O}\overset{k_{+}}{\underset{k_{-}}{⇌}}{\text{H}}^{+}+{\text{HC}{\text{O}}_{3}}^{-},\;\;\text{C}{\text{O}}_{2}+\text{O}{\text{H}}^{-}\overset{k_{2}}{\underset{k_{-2}}{⇌}}{\text{HC}{\text{O}}_{3}}^{-}, \end{array}$$

where *k*_+_,  *k*_−_,  *k*_2_, and *k*_−2_ are their reaction constants with their equilibrium constants defined as *K*_1_ and *K*_2_, respectively. The overall reaction is

(20) $$\begin{array}{c} \text{C}{\text{O}}_{2}+{\text{C}{\text{O}}_{3}}^{2-}+{\text{H}}_{2}\text{O}⇌2{\text{HC}{\text{O}}_{3}}^{-}, \end{array}$$

with the following fast reactions assumed to be at equilibrium

(21) $$\begin{array}{c} {\text{HC}{\text{O}}_{3}}^{-}\overset{K_{3}}{\underset{}{⇌}}{\text{H}}^{+}+{\text{C}{\text{O}}_{3}}^{-},\;\;{\text{H}}_{2}\text{O}\;\;+\overset{K_{4}}{\underset{}{⇌}}{\text{H}}^{+}+\text{O}{\text{H}}^{-}, \end{array}$$

where *K*_3_ and *K*_4_ are their equilibrium constants. The parameters and their values are stated in Table 1.

### 2.4. Dialysate-Side Flow

**Figure 2:**
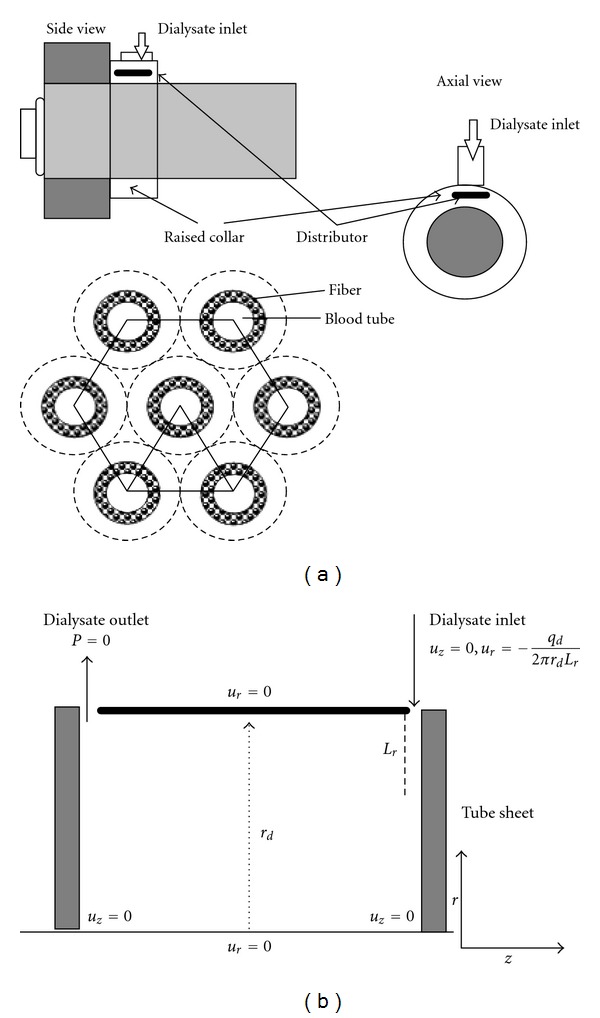
Geometric configuration near dialysate inlet. (a) The distributors are designed to keep the dialysate entering the hollow-fibers uniformly. (b) Schematic computational domain at the dialysate compartment.

Since each fiber was surrounded by a uniform annulus (shown in Figure 2(a)), we adapted Krogh cylinder geometry [36, 37] with annulus radius *r*_*d*_ which was far larger than the fiber radius *r*_*b*_. Assuming a fully developed axial and radial velocities in annulus geometry, the generic continuity and momentum equations reduced to (22) as reported by [38], with specified boundary conditions of *u*_*z*_ = 0 at *r* = 0 and *r* = *r*_*b*_

(22) $$\begin{array}{c} u_{r}\left(r_{b}\right)=-\frac{q_{d}}{2\pi r_{b}L_{r}},\;\forall r_{b},\\ u_{z}=\frac{2q_{d}}{\pi r_{d}^{2}L_{r}}\frac{\ln\left(r/r_{d}\right)-\left({\left(r/r_{d}\right)}^{2}-1\right)/\left(\kappa^{2}-1\right)\;\ln\left(\kappa \right)}{\left(\kappa^{2}+1\right)\ln\left(\kappa \right)+1-\kappa^{2}}. \end{array}$$

Here, the parameter *q*_*d*_ represented flow rate in the dialysate inlet, *κ* the ratio of *r*_*b*_/*r*_*d*_, and *L*_*r*_ the width of the raised collar used to promote uniform flow in dialyzers. The solute replenishment term, *R*_*s*_, was introduced to help maintain dialysate concentration level and was calculated using [31]

(23) $$\begin{array}{c} R_{s}=\varepsilon c_{s}\left(c_{s_{0}}-c_{s}\right), \end{array}$$

where *ε* is the replenishment coefficient.

The transport of solutes in the annulus, shown in Figure 2(b), involving convection and diffusion with *u*_*r*_  and  *u*_*z*_ defined by (22) could be described similarly as (16) as

(24) ∂cs∂t︷Transient+  uz∂cs∂z+ur∂cs∂r︷Convective    =  Ds(∂2cs∂r2+1r∂cs∂r+∂2cs∂z2)︷Diffusive    +Rs︷Replenishment.

## 3. Algorithm and Numerical Techniques

Finite volume method (FVM) was used to transform the model equations (12)–(24) into dimensionless system. Since the structure of the convection-diffusion equations (16) and (24) only differed by the source term, we replaced the source term by *ψ*. 

### 3.1. Transformation of Models Using FVM

Integrating both sides of (16) or (24) over a small control volume *CV* gave

(25) ∂∂t∫CVcsdV+∫CVuz∂cs∂zdV  =∫CVDs{∂2cs∂r2+1r∂cs∂r+∂2cs∂z2}dV+∫CVψdV.

Thus, (25) means that the rate of increase of concentration with time in the volume element is equal to the convective flow into the volume element, plus the diffusive flow, and the creation of new solute from the source term totaled over the volume element. Since both convective and diffusive terms represented divergence of vector fields (i.e., the fluid flow vector and the concentration gradient, resp.), we applied the divergence theorem to the integrals of these terms to convert them to surface integrals. 

#### 3.1.1. Convective Term

Since the flow velocity $\overset{-}{u}$ is only in the *z*-direction, the convective flow $c_{s}\overset{-}{u}$ is

(26) $$\begin{array}{c} c_{s}\overset{-}{u}=\left[\begin{array}{c} 0\\ c_{s}u_{z}\left(r\right) \end{array}\right]. \end{array}$$

Therefore, the divergence of the convective flow vector $c_{s}\overset{-}{u}$ using cylindrical coordinate is

(27) div⁡(csu−)=1r∂(rcsur)∂r+1r∂csuθ∂θ+∂csuz∂z=∂csuz∂z=∂cs∂zuz+cs∂uz∂z=∂cs∂zuz,

where we have used the fact that the flow components in the *r* and *θ* directions are zero and that the *z* component is a function of *r* only, implying ∂*u*_*z*_/∂*z* = 0. Thus, the second term on the left-hand side of (25) is

(28) $$\begin{array}{c} \int_{CV}^{}u_{z}\frac{\partial c_{s}}{\partial z}dV=\int_{CV}^{}\mathrm{div}\left(c_{s}\overset{-}{u}\right)dV=\oint_{A}^{}\left(\overset{-}{n}\cdot \left(c_{s}\overset{-}{u}\right)\right)dA. \end{array}$$

Since the vector $c_{s}\overset{-}{u}$ is in the *z*-direction, it does not cross the surfaces of the control volume cube on the faces in *r* and *θ* directions. That is, the normal to those faces are perpendicular to the flow vector and so the dot product is zero. Therefore, the only nonzero parts of the surface integral are those over the faces in +*z* and −*z* directions of the cube. The normal to those faces is parallel to the convection vector (in the +*z* direction and antiparallel in the −*z* direction), so the integral becomes

(29) ∮A(n−·(csu−))dA=∮+zcs(r,zc+Δz2)uz(r)r dr dθ−∮−zcs(r,zc−Δz2)uz(r)r dr dθ,

where Δ*z* is the size of the volume cube in the *z*-direction and *z*_*c*_ is the *z* coordinate at the center of the cubic volume. In the process of discretization, we approximate the values of *c*_*s*_ and *u*_*z*_ over the surface area of the cube by their values at the nearby grid points as *c*_*s*_*a*__(*r*, *z*) and *u*_*s*_*a*__(*r*), respectively, if the grid point is sufficiently fine. Thus,

(30) ∮A(n−·(csu−))dA  =csa(r,zc+Δz2)uza(r)∮+zr dr dθ   −csa(r,zc−Δz2)uza(r)∮−zr dr dθ,  =uza(r)[csa(r,zc+Δz2)−csa(r,zc−Δz2)]ΔAz.

#### 3.1.2. Diffusive Term

Since diffusion is driven by concentration gradient, the divergence vector field in cylindrical coordinates is

(31) div⁡(grad(c))=1r∂∂r(r∂c∂r)+∂∂z(∂c∂z),=1r[1·∂c∂r+r·∂2c∂r2]+∂2c∂z2,=1r∂c∂r+∂2c∂r2+∂2c∂z2.

Using the divergence theorem, the diffusion term in (25) could be written as

(32) $$\begin{array}{c} D_{s}\int_{CV}^{}\mathrm{div}\left(\text{grad}\left(c_{s}\right)\right)dV=D_{s}\oint_{A}^{}\left(\overset{-}{n}\cdot \left[\begin{array}{c} \frac{\partial c_{s}}{\partial r}\\ \frac{\partial c_{s}}{\partial z} \end{array}\right]\right)dA. \end{array}$$

For the surface integral on the +*r* and −*r* faces, only the first element of the gradient vector is applicable (the normal to those faces picks out that component of the vector) while the second element is used on the +*z* and −*z* faces only. As a result, the integral (32) becomse

(33) Ds∮A(n−·[∂cs∂r∂cs∂z])dA =Ds[{∮+r∂cs(rc+Δr/2,z)∂rdAr−∮−r∂cs(rc−Δr/2,z)∂rdAr}   +{∮+z∂cs(r,zc+Δz/2)∂zdAz     −∮−z∂cs(r,zc−Δz/2)∂zdAz}].

Approximating the values of ∂*c*_*s*_/∂*r* and ∂*c*_*s*_/∂*z* over the face area by indicating their values with subscript “*a*” and pull the constant values out of the integral, right-hand side of (33) becomes

(34) Ds∮A(n−·[∂cs∂r∂cs∂z])dA=Ds[ΔAr{∂csa(rc+Δr/2,z)∂r−∂csa(rc−Δr/2,z)∂r}   +ΔAz{∂csa(r,zc+Δz/2)∂z−∂csa(r,zc−Δz/2)∂z}].

Thus, the LHS of (25) with ${\overset{-}{c}}_{s}$ defining the volume average of solute concentration is

(35) 1ΔV∫CV∂cs∂tdV  +1ΔV∫CVuz∂cs∂zdV=∂∂t(1ΔV∫CVcsdV)  +uza(r)ΔAzΔV[csa(r,zc+Δz2)−csa(r,zc−Δz2)] =∂c−s∂t+uza(r)[csa(r,zc+Δz/2)−csa(r,zc−Δz/2)]Δz.

The RHS of (25) becomes

(36) 1ΔV∫CVDs{∂2cs∂r2+1r∂cs∂r+∂2cs∂z2}dV+1ΔV∫CVψdV =(DsΔV)  ×[ΔAr{∂csa(rc+Δr/2,z)∂r−∂csa(rc−Δr/2,z)∂r}    +ΔAz{∂csa(r,zc+Δz/2)∂z        −∂csa(r,zc−Δz/2)∂z}]+ψ−, =Ds[1Δr{∂csa(rc+Δr/2,z)∂r−∂csa(rc−Δr/2,z)∂r}    +1Δz{∂csa(r,zc+Δz/2)∂z        −∂csa(r,zc−Δz/2)∂z}]+ψ−,

where $\overset{-}{\psi }=\left(1/\Delta V\right)\int_{CV}^{}\psi dV$.

### 3.2. Scaling to Dimensionless Form

The transformed equations (35) and (36) and their initial and boundary conditions (14)-(15) and (17)–(23) were converted into nondimensional forms using the same scale factors for both blood and dialysate flow regions. The nondimensional variables used in the transformation are indicated with superscript “*” below and the reference variables defined in Table 2

(37) r∗=rL;  z∗=zL;  ur∗=urU;uz∗=uzU;  A1=k+LU;  Shs=LPsDs;cs∗=cscs0;  t∗=tUL;  ϕ=rbL;  Pe=LUDs;  Re=ρUrbμ;  Es=LpLΔp2Ds;

where Pe and Sh are Pe'clet and Sherwood numbers, respectively, and the ratio of momentum diffusivity and mass diffusivity is donated by *E*. Pe = *LU*/*D*_*s*_ = *q*_*b*_/*πϕr*_*b*_ *D*_*s*_ expressed the relative importance of convection to diffusion while *Re* = *ρUr*_*b*_/*μ* = *ρq*_*b*_/*πμr*_*b*_ related inertial effects to viscous effects. Since dialysis devices employ laminar fluids flow with *Re* ≪ 1 the inertial effects would be irrelevant [31, 41].

Substituting the dimensionless variables in (37) into (35) and (36), simplifying notations, and dropping the superscript “*” resulted in

(38) ∂c−s∂t+uza(r)[csa(r,zc+Δz/2)−csa(r,zc−Δz/2)]Δz=(1Pe)[1Δr{∂csa(rc+Δr/2,z)∂r−∂csa(rc−Δr/2,z)∂r}     +1Δz{∂csa(r,zc+Δz/2)∂z−∂csa(r,zc−Δz/2)∂z}] +(LUcs0)ψ−(r,z).

The dimensionless initial and boundary conditions, buffer and replenishment, and membrane interfacial conditions corresponding to (38) were as follows.

*Blood-Side Inlet Velocity Conditions*

(39) $$\begin{array}{c} u_{r}=0,\;\;u_{z}=\frac{2}{N}\left(1-\frac{r^{2}}{\phi^{2}}\right). \end{array}$$

*Dialysate-Side Inlet and Outlet Velocities*

(40) ur=−rd2κLr ∀rbuz=2Lr(ϕ2(κ2−1)ln⁡⁡(κ)·[ln⁡(κr)−ln⁡(ϕ)]−[(κr)2−ϕ2]ϕ2[(κ4−1)ln⁡(κ)−(κ2−1)2]ln⁡⁡(κ))

*No Slip and Axisymmetric Conditions*

(41) $$\begin{array}{c} u_{r}=u_{z}=0,\;u_{r}=\frac{\partial u_{z}}{\partial r}=0\;\text{at}\;\;r=0;\;\;0\leq z\leq 1. \end{array}$$

*Inlet and Outlet Blood and Dialysate Concentrations*

(42) $$\begin{array}{c} c_{s}\left(z,r,0\right)=1,\;\;c_{s}\left(0,r,t\right)=1,\;\;\frac{\partial c_{s}\left(z,0,t\right)}{\partial r}=0. \end{array}$$

*Buffer and Replenishment Terms*

(43) $$\begin{array}{c} {\overset{-}{\psi }}_{\text{C}{\text{O}}_{2}}=-\frac{A_{1}}{c_{s_{0}}}\left(\left[\text{C}{\text{O}}_{2}\right]-20\beta \right)\left(1+0.1\alpha \right),\;\;{\overset{-}{\psi }}_{{\text{HC}{\text{O}}_{3}}^{-}}=-2{\overset{-}{\psi }}_{\text{C}{\text{O}}_{2}},\\ \psi_{R_{s}}=\frac{\varepsilon c_{s_{0}}L}{U}c_{s}\left(1-c_{s}\right), \end{array}$$

where ${\overset{-}{\psi }}_{\text{C}{\text{O}}_{2}}\;\text{and}\;{\overset{-}{\psi }}_{{\text{HC}{\text{O}}_{3}}^{-}}$ represented dimensionless buffer terms in blood side and ${\overset{-}{\psi }}_{R_{s}}$ depicted dimensionless replenishment term for solute *s* = CO_2_ and HCO_3_^−^.

*Blood-Membrane Interfacial Conditions*

(44) uz=0,  ur=JvU=EsPes,∂cs∂r=Es(1−Cs∗)−Shs·Δcs,

where Sh_*s*_ is the Sherwood number and *E* is the ratio of momentum and mass diffusivity defined in (37). 

### 3.3. Model Parameters and Numerical Algorithm 

#### 3.3.1. Geometric and Transport Parameters

The hollow-fiber dialyzer chosen for this study was the Fresenius' F60 model with membrane area was 1.15 m^2^. The membrane module has 22 cm effective axial length with 200 *μ*m and 40 *μ*m fiber diameter and thickness, respectively [20]. The initial inlet bicarbonate concentration values of blood and dialysate were set to 19 mol·m^−3^ and 35 mol·m^−3^, respectively, while the blood-side and dialysate-side flow rates were, respectively, 400 mL/min (i.e., 6.65  ×  10^−6^ m^3^ s^−1^) and 800 mL/min (1.33 × 10^−5^ m^3^ s^−1^) [31, 42]. Other parameters and constant values used in this paper are either listed in Table 3 or are computed using values in Table 3.

#### 3.3.2. Variables and Grid Definition

Application of FVM resulted in the creation of grid structured such that the number of rectangular cells in *r* and *z* direction remained constant throughout the domain of interest. For the spatial domain, the numerical model used separate subdomain grids for the blood side and the dialysate side since the two models and their domain dimensions were different. The spatial grid had a variable number of intervals in each axis, defined by the variables *R*_*b*max⁡_, *R*_*d*max⁡_, and *R*_max⁡_. Because of the FVM development, the boundaries of the domain of interest have to be at the faces of the rectangular control volumes, rather than at a grid point. Thus, for example, the inlet boundary condition applied at *z* = 0, so the first internal grid point is at 0 + *z*_grid_/2, where *z*_grid_ defined the grid spacing. However, one extra grid row or column outside the boundary was used to allow easy application of boundary conditions. Thus, the first grid point in *z* was outside the inlet at *z* = −*z*_grid_/2. Therefore, the grid size in each axis was

(45) zgrid=1zmax⁡−2,  Rb grid=RbRa(Rbmax⁡−2),Rd grid=RdRa(Rdmax⁡−2),

since the domain of interest in the model was 0 < *z* < 1 for the *z*-direction and 0 < *r* < *r*_*b*_/*L* and 0 < *r* < *r*_*d*_/*L* in the *r*-direction for blood and dialysate sides, respectively.

The indices of the variables (such as solute concentration) ran from *i* = 1 to *z*_max⁡_ and *j* = 1 to *R*_*b*max⁡_ or *R*_*d*max⁡_. Therefore, the boundaries of the spatial domains were between indices *j* = 1 and 2 and between *j* = *R*_*b*max⁡_ − 1 and *R*_*b*max⁡_ (blood side), similarly for the dialysate side, it was between *i* = 1 and 2 and between *i* = *z*_max⁡_ − 1 and *i* = *z*_max⁡_. Since the boundary conditions define the values of the variables there, the numerical model only calculated the values in the range from 2 to *z*_max⁡_ − 1 and so on. 

For the time coordinate, a time increment of *dt* was used to sample the time axis. The real time represented by a sample is *t*(*k*) = (*k* − 1)*dt*, *k* = 1,2,…. Finally, we considered two types of solutes defined by the subscript *s* in the models, as per the Table 4.

Therefore, the variables in the domain of interest could be represented by a 4-dimensional array *x*(*i*, *j*, *k*, *s*) where the variable *x* can be *r*, *z*, *t*, *c*, *u*, *B*, and *R*. 

#### 3.3.3. Hybrid Differencing Scheme

The continuous diffusion terms in (38) were numerically discretized using the hybrid differencing scheme. The scheme switched to the upwind differencing when the central differencing produced inaccurate results at high Peclet numbers. Also, since the partial continuous derivatives were at the faces of the control volume, we used the values at the center and adjacent grid points to find the values of ∂*c*_*s*_/∂*z* and ∂*c*_*s*_/∂*r*. The convection terms in (38) used the upstream scheme to estimate the value of the function at the control volume face. Thus, the functional values at the faces of our model system were represented by the following. 

Blood Side:

(46) csa(r,zc+Δz2)=cb(r,zc)=cb(i,j,k+1,s),csa(r,zc−Δz2)=cb(r,zc−Δz)=cb(i−1,j,k+1,s).

Dialysate Side:

(47) csa(r,zc+Δz2)=cd(r,zc+Δz)=cd(i+1,j,k+1,s),csa(r,zc−Δz2)=cd(r,zc)=cd(i,j,k+1,s).

#### 3.3.4. Boundary Conditions and Stability

**Figure 3:**
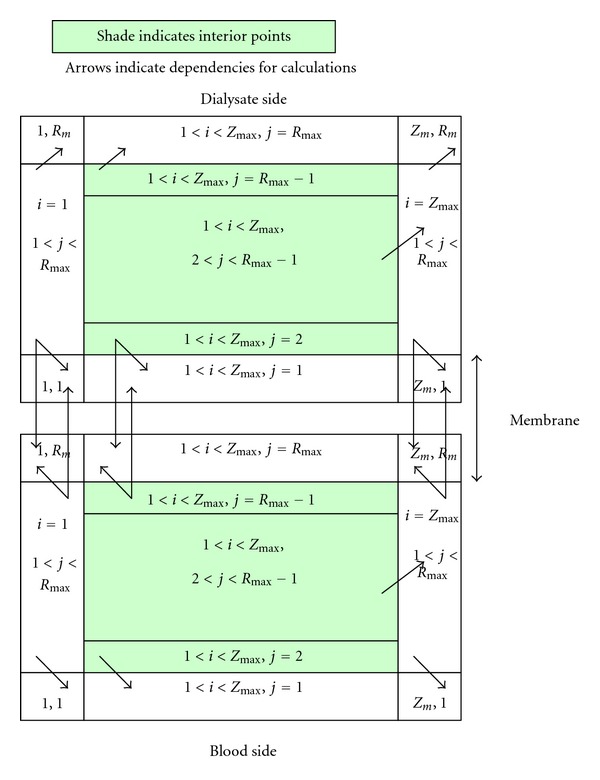
Dependencies between the BCs with arrows, which in turn defined the sequence of application required by these dependencies. The BC at the upstream boundary in each side was a constant and all other BCs depended on values in the current time step [31].

Since many of the boundary conditions (BCs) depended on values in adjacent grid points, and the numerical equations for our model system only defined the values in the interior of the domain, the BCs were defined in terms of grid values to the interior of that boundary. Therefore, the corners were resolved by simulating the interior points of the model system followed by the BCs of the blood, dialysate, and the membrane (see Figure 3).

## 4. Results and Discussions

The numerical solution presented in the previous sections allowed us to determine the concentration gradients, convective and diffusive fluxes, total flux, and concentrations of carbon dioxide and bicarbonate profiles for high-flux membrane. Diffusive solute transport across the membrane was predominantly driven by concentration gradients, whereas convection transport was determined by pressure gradients.

### 4.1. Carbon Dioxide and Bicarbonate Concentration Gradients

**Figure 4:**
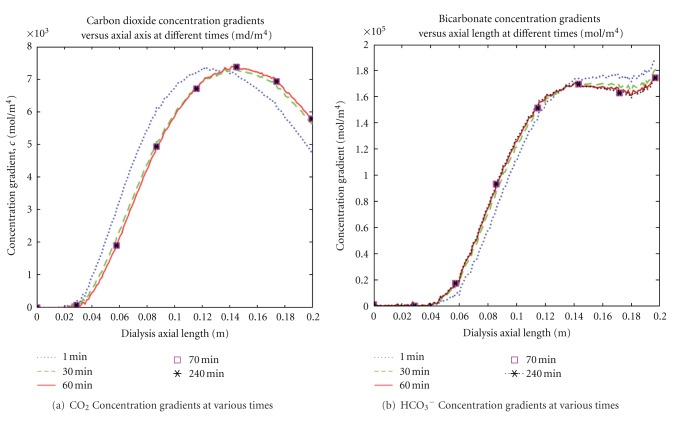
Bicarbonate and carbon dioxide concentration gradients increased along the membrane axial distance at different time periods. (a) Depicted carbon dioxide maxima gradients shifting toward the membrane end and decreasing from their maxima. (b) Displayed bicarbonate gradients peaking at the same position inside the membrane. Maxima concentration gradients were achieved for both carbon dioxide and bicarbonate under an hour during the dialysis therapy.

Concentration gradient has been the principal process for removing end-products of metabolism (urea, creatinine, uric acid) and for repletion of bicarbonate deficit of metabolic acidosis associated with end-stage renal disease patients.  Figure 4 showed the results of the concentration gradient profiles for carbon dioxide and bicarbonate solutes in the membrane for different time periods. The membrane carbon dioxide concentration gradient profiles (see Figure 4(a)) increased, reaching maxima gradients inside the membrane, then appeared to diminish from their maxima along the dialyzer axial length. The maximum points do shift toward the end of the membrane length while the maximum magnitude occurred at *t* = 70 minutes. 

Figure 4(b) depicted positive increased bicarbonate concentration gradients at different time periods in the membrane as the axial distance increased. The gradients peaked around the same position at *t* = 60 and appeared to experience reduction of the bicarbonate concentration gradients toward the end of the membrane. Since bicarbonate containing dialysate was used in our model, it was important to have adequate concentration gradients to generate bicarbonate flux into the blood to restore body buffer. The adequacy of bicarbonate concentration gradient was observed as shown in Figure 4(b).

### 4.2. Carbon Dioxide and Bicarbonate Diffusive Fluxes

**Figure 5:**
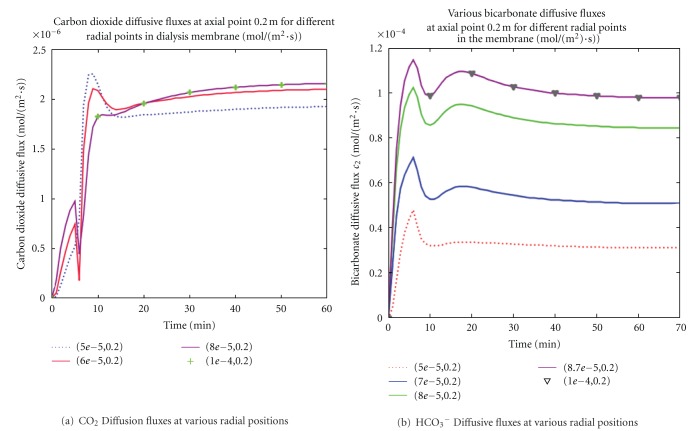
Bicarbonate and carbon dioxide diffusive fluxes in the membrane at dialyzer axial distance *z* = 20 cm. Diffusive solute fluxes increased as the radial distance increased. Also, diffusion for both solutes was stronger near the fiber walls than the center. The carbon dioxide and bicarbonate ions effect observed before solute stability could enhance the diffusion process during dialysis session.

The fluxe profiles for carbon dioxide and bicarbonate in the membrane were presented at the membrane region (*z* = 20 cm) for different time periods in Figure 5.  Both 5(a) and 5(b) showed the unsteady characteristics of solute diffusive fluxes at various radial positions in the membrane. In all the chosen radial locations, the rate of mass transfer of the CO_2_ and HCO_3_^−^ solutes increased at the onset of the dialysis therapy followed by a small fluctuations and then became constant during the remaining therapy session. In Figure 5(a), the sharp increased in CO_2_ may be caused by (i) the active hydrogen ion from the blood reacting with bicarbonate ions to produce more of the CO_2_ and/or (ii) the incomplete dissociation of bicarbonate ion into CO_2_ in the membrane. Similarly, the initial bicarbonate sharp increase observed may be explained by the incomplete dissociation of carbonic acid into bicarbonate and hydrogen ions. These observations suggested a bicarbonate ion carryover effect in the membrane during dialysis therapies. In addition, both figures depicted increased diffusive fluxes toward the membrane end and an abnormally high solute fluxes near the blood-membrane interface. This abnormality may increase the driving force for diffusion and eventually enhance the diffusive process during dialysis session.

### 4.3. Carbon Dioxide and Bicarbonate Convective Fluxes

**Figure 6:**
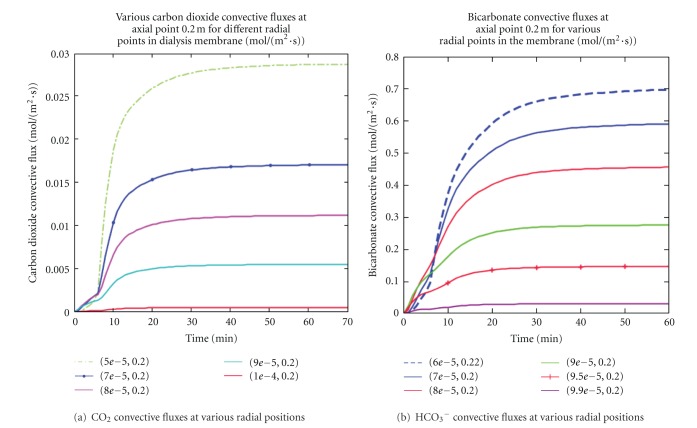
Various carbon dioxide and bicarbonate convective fluxes at different radial distances were presented. Contrary to diffusive flux, convective flux decreased at the fiber wall and increased in the fiber center. Also, the sagging nature of convective fluxes observed at the blood port for the solutes may explain the high diffusive flux abnormalities observed in Figure 5.

Figure 6 showed the results of carbon dioxide and bicarbonate convective solute fluxes in the dialyzer membrane for various radial positions in the first hour of dialysis session. Compared to Figure 5, Figure 6 displayed the dominance of convective fluxes for small solutes (CO_2_ and HCO_3_^−^) during a high-flux hollow-fiber dialysis session. The magnitude of CO_2_ and HCO_3_^−^ fluxes were determined by the natural convection (transmembrane pressure gradient) and the forced convection (mass inflow). Both figures also indicated that convective fluxes decreased in the membrane with increased radial distance as one moved toward the end of the axial length. Thus, in this model there was a continuous interference between convective and diffusive fluxes (see Figures 5 and 6). In this case, increasing one type of transport mechanism would decrease the other and therefore could be beneficial or detrimental on dialyzer's efficiency. In the region near the blood ports, the sagging nature of the convective profiles may explain the abnormally high diffusive flux of solutes at the blood-membrane interface observed in Figure 5. This observation craves the need to seek minimal interference between convection and diffusion during dialysis therapy.

### 4.4. Total Fluxes in Dialysis Membrane

**Figure 7:**
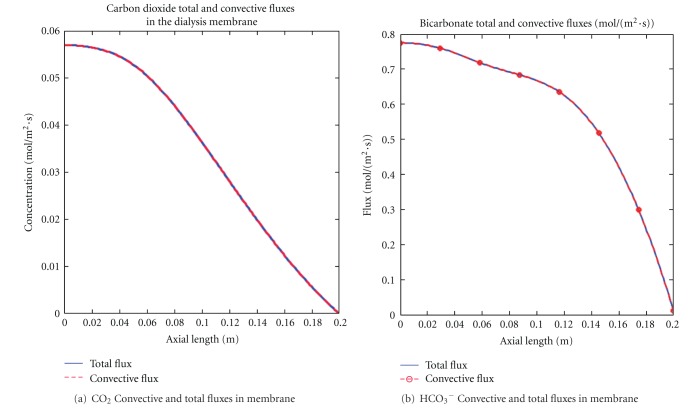
Total fluxes for both carbon dioxide and bicarbonate solutes were mediated by convective fluxes. Total fluxes decreased along the dialyzer distance.

Figure 7 displayed the dominance of convective flux over diffusion when a high-flux hollow-fiber dialyzer was used. Both figures showed that the total flux (convection and diffusion) of solutes were mediated by convective flux and it decreased along the axial length. The decreasing profile within the membrane may be explained by the decreasing nature of the transmembrane pressure gradient or the solute accumulation at the membrane surface over time. Thus, in addition to convection playing a major role in higher molecular weight solute transports [13], it was also more efficient than diffusion in small solutes transport when high-flux dialysis membrane was used. 

### 4.5. Carbon Dioxide Concentration

**Figure 8:**
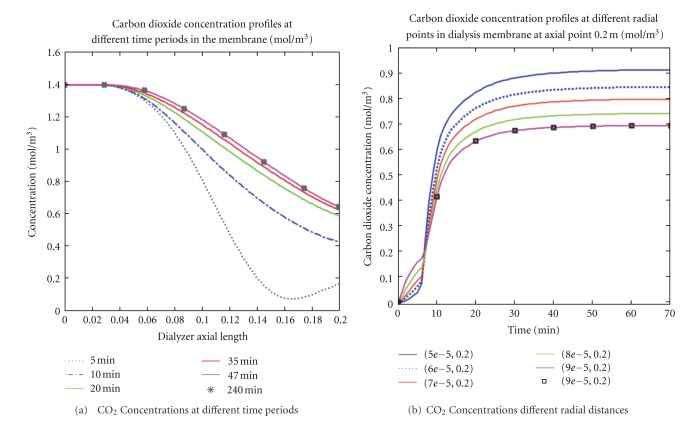
The effects of diffusion and convection fluxes and concentration gradients for carbon dioxide solute at different time periods and radial distances were presented. Carbon dioxide decreased over time implying carbon dioxide solute desorption in the membrane (a). Also, carbon dioxide concentration was higher in the fiber center than near the fiber walls (b).

Carbon dioxide concentration profiles in the membrane at various time periods and radial distances during high-flux dialysis session were shown in Figure 8. In Figure 8(a), various CO_2_ concentration profiles at different time periods were shown as a function of membrane axial distance. The result clearly showed that the CO_2_ concentration decreased as the axial length increased and that most CO_2_ desorption occurred within 47 minutes of the therapy session. The constant profiles after 47 minutes indicated that the membrane was fully saturated with CO_2_ concentration during the therapy. In Figure 8(b), CO_2_ concentration profiles as a function of the first 70 minutes of dialysis session was shown at different radial positions. The result demonstrated that CO_2_ concentration increased as one moved toward the end of the membrane. Also, the CO_2_ concentration near the wall of the fiber was much higher than that of the center of the fiber at the same axial position. At the outlet, the concentration was stable with respect to radial position, indicating CO_2_ saturation in the membrane. 

### 4.6. Bicarbonate Concentration

**Figure 9:**
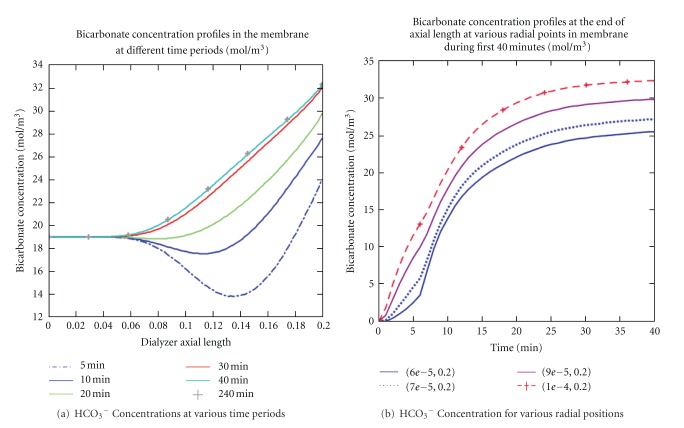
Bicarbonate concentration profiles at various time periods and radial distances in the membrane were presented. At different time periods, bicarbonate concentration increased rapidly for during the first 30 minutes and then slowly for another 10 minutes, before becoming stable for the rest of the therapy (a). The maximum concentration reached was within the physiologic range reported by clinical studies. In (b), the bicarbonate concentration profiles within the first 40 minutes at various radial distances were found to increase until the membrane becoming nearly saturated with the solute. However, concentration at the fiber walls was much higher than that in the fiber center.

Similarly, bicarbonate concentration profiles in the membrane at various time periods and radial positions during high-flux dialysis session were shown in Figure 9. At various time periods, the result indicated a HCO_3_^−^ concentration increased as the membrane axial distance increased (see Figure 9(a)). Also, it was shown that HCO_3_^−^ concentration increased rapidly within the first 30 minutes and then became stable after 40 minutes of the therapy session. In Figure 9(b), the HCO_3_^−^ concentration profiles at axial distance (*z* = 0.20 m) against time for various radial distances was shown. HCO_3_^−^ concentration increased and was nearly saturated in the membrane. The rate of increased and the degree at which HCO_3_^−^ concentration increased may be determined by the immediate buffer response through convection and diffusion and the extent to which organic acid production in the membrane is increased. In addition, these observations confirmed clinical studies [31, 42] that dialysis patients achieve stable physiologic HCO_3_^−^ concentration levels during dialysis therapy.

## 5. Conclusions

A mathematical model that coupled nonlinear unsteady convection-diffusion mass transfer of small solutes in high-flux dialyzer with buffer during dialysis session was developed. Finite volume technique was used to transform the model equations and numerical discretization and analysis schemes were then proposed and tested. The solute concentration gradients, diffusive and convective fluxes, and their effects on overall concentrations were explored. The salient observations were summarized as follows:

 Both carbon dioxide and bicarbonate concentration gradients increased and reached maxima gradient values inside the dialyzer membrane. While CO_2_ concentration gradients appeared to diminish from their maxima and shift toward the end of the membrane, HCO_3_^−^ concentration gradients peaked at the same position. In addition, the magnitude of the HCO_3_^−^ concentration gradient was large enough to activate HCO_3_^−^ diffusion in the membrane.
 Diffusive fluxes for carbon dioxide and bicarbonate showed increased profiles at different radial distances in the membrane. Abnormally high fluxes were observed near the blood-membrane interface that could increase diffusion driving force and eventually enhance the overall diffusive process during dialysis session.
 Convective flux still dominated total flux for small solute (CO_2_ and HCO_3_^−^) transfer during high-flux dialysis therapy. However, the continuous interference between convective and diffusive fluxes could be beneficial or detrimental when accessing high-flux dialyzer efficiency for small solute transport.
 Carbon dioxide concentration decreased rapidly causing the membrane to become fully saturated with CO_2_ within 47 minutes. Further investigation showed an increased CO_2_ concentration towards the end of the membrane which indicated higher CO_2_ concentration near the walls of the fiber than the fiber center at the same axial distance.
 Similarly, there was an optimal HCO_3_^−^ concentration in the membrane within 30 minutes of dialysis therapy because of the effects of convection and the preponderance of diffusion. The rate of increased and the degree at which bicarbonate increased could be caused by immediate buffer response and the positive effect of convection on diffusion or the extent to which organic acid production in the membrane was increased.

Therefore, the model presented provided an accurate quantitative description of both convection and diffusion through high-flux membrane with buffer. This is critical for the effective design and efficient operation of dialyzers.

## Figures and Tables

**Table 1:** Reaction and equilibrium constants and equations used in this paper at 297 K.

Constant	Value/equation	Unit	Ref
Forward reaction constant, *k*_+_	2.38 × 10^−2^	s^−1^	[31, 32]
Reverse reaction constant, *k*_−_	1.4 × 10^1^	m^3^ mol^−1^ s^−1^	[31, 33]
Forward reaction constant, *k*_2_	8.67	m^3^ mol^−1^ s^−1^	[31, 32]
Reverse reaction constant, *k*_−2_	2.0 × 10^−4^	s^−1^	[31, 32]
Equilibrium constant, *K*_1_	4.43 × 10^−4^	mol m^−3^	[32]
Equilibrium constant, *K*_2_	4.905 × 10^4^	mol^−1^ m^3^	[32]
Equilibrium constant, *K*_3_	4.64 × 10^−8^	mol m^−3^	[32]
Equilibrium constant, *K*_4_	9.03 × 10^−9^	mol^2^ m^−6^	[32]

**Table 2:** The reference variables with their description.

Symbol	Description
*L*	Reference length: they are the same for both compartments
*U*	Reference velocity
*c*_*s*_0__	Reference solute concentration

**Table 3:** Geometric and transport characteristics of the hollow-fiber module used.

Parameter (unit)	Notation	Value	Ref.
Diffusion coefficient of CO_2_ in blood (m^2^ s^−1^)	*D*_CO_2_,*b*_	3.4 × 10^−10^	[31–33, 35]
Diffusion coefficient of HCO_3_ in blood (m^2^ s^−1^)	*D*_HCO_3_,*b*_	1.4 × 10^−10^	[31–33, 35]
CO_2_ diffusion coefficient in dialysate (m^2^ s^−1^)	*D*_CO_2_,*d*_	1.59 × 10^−9^	[31–33, 35]
HCO_3_^−^ diffusion coefficient in dialysate (m^2^ s^−1^)	*D*_HCO_3_,*d*_	1.18 × 10^−9^	[31–33, 35]
Membrane effective length (m)	*L*	0.20	[31]
Hydraulic permeability (m/s Pa)	*L*_*p*_	1.15 × 10^−10^	[31]
Width of raise collar (m)	*L*_*r*_	0.014	[31]
Fiber diameter (*μ*m)	*L*_*f*_	200	[31, 39]
Fiber thickness (*μ*m)	*e*	40	[40]
Number of fibers	*N*	9000–12000	F60 Model
Membrane permeability of CO_2_ (m s^−1^)	*P*_CO_2__	1.72 × 10^−9^	[32, 33]
Membrane permeability of HCO_3_ (m s^−1^)	*P*_HCO_3_^−^_	1.95 × 10^−9^	[32, 33]
Radius of dialysate channel (m)	*r*_*d*_	1.25 × 10^−4^	[39, 40]
Radius of blood channel (m)	*r*_*b*_	2.0 × 10^−4^	[39, 40]
Initial velocity at blood inlet (m s^−1^)	*u*_*b*_	1.73 × 10^−2^	[31, 39, 40]
Initial velocity at dialysate inlet (m s^−1^)	*u*_*d*_	1.21 × 10^−2^	[31, 39, 40]

**Table 4:** Solutes indexes for numerical computations.

Value for *s* index	Solute type
1	CO_2_ (carbon dioxide)
2	HCO_3_^−^ (bicarbonate)
